# Supplementary material for: A Fast, Flexible Simulation Framework for Bayesian Adaptive Designs—The R Package BATSS
Source: Pharm Stat. 2026 Aug 10;25(5):e70109. doi: 10.1002/pst.70109 (PMC13456979; doi:10.1002/pst.70109)
Supplement: Supplementary file 1 [file PST-25-0-s001.pdf]

Appendix C
Binary outcome case study
Full code and output

July 7, 2026

1 Case Study 2.1 - "Alternative" scenario

Here we first provide a description of the trial and then provide the R code and output for Case Study 2.1 from the main manuscript (with a fixed cut-off for the futility stopping boundary) for the "Alternative" scenario.

1.1 Case Study 2 – Trial description

This case study is motivated by the Personalised Immunotherapy Platform (PIP) trial (?), a phase II randomised controlled trial currently under development at the Melanoma Institute Australia. The PIP trial aims to evaluate biomarker-driven treatment selection of novel immunotherapy combinations against standard combination checkpoint inhibitor therapy in patients with advanced melanoma who are predicted to be unlikely to respond to standard first-line immunotherapy treatments. The trial compares five novel drug combinations (arms "B" to "F") against standard of care (arm "A"), with the primary outcome being the objective response rate (ORR) at 6 months post-randomisation according to RECIST criteria. The aim is to determine which, if any, of the novel combinations are superior to the standard of care, via pairwise comparisons of each intervention arm to control; head-to-head comparisons between the novel combinations are not of primary interest. Refer to Section 4 of the main manuscript for a description of the design parameters used in our simulation.

In our case study, we consider five novel drug combinations (arms "B" to "F") compared against standard of care (arm "A").

1.2 Case Study 2.1 Code

```
library(BATSS)

# assumed true ORR for each arm:
p0=.40; p1=.40; p2=.40; p3=.50; p4=.70; p5=.70
# beta parameters on log-OR scale:
bb0=log(p0/(1-p0)) #control arm / intercept
bb1=log((p1/(1-p1))/(p0/(1-p0))) #arm B tr effect
bb2=log((p2/(1-p2))/(p0/(1-p0)))
bb3=log((p3/(1-p3))/(p0/(1-p0)))
bb4=log((p4/(1-p4))/(p0/(1-p0)))
bb5=log((p5/(1-p5))/(p0/(1-p0))) #arm F tr effect

# calculation of delta.futility for a 10% absolute improvement over control
pp=p0+0.1
delta.f=log((pp/(1-pp))/(p0/(1-p0)))

# specify group allocation function. Note that we use this function,
# rather than the one specified in the main manuscript,
# as it leads to allocations that more closely match the intended randomisation
# probabilities.
# Note also that this function corresponds to the function alloc.balanced
# available in the BATSS package.
group.fun= function(m, prob){
  prob = abs(prob)/sum(abs(prob)) #normalise probabilities
  m0.g = floor(prob*m)
  m0   = sum(m0.g)
  factor(rep(names(prob),m0.g+rmultinom(1,m-m0,prob)),
         levels=names(prob))
}

# specify function to declare efficacy for an arm
# stopping for efficacy only occurs at the last look (maximum sample size)
efficacy.arm.fun = function(posterior, b.eff){
   posterior > 1-b.eff
}
# where delta.eff needs to have NAs to not evaluate efficacy at interims

# specify function to declare futility for an arm (using a fixed value)
# Note that this function corresponds to the fut.arm.simple function
# that is available in the BATSS package, so instead you could
# specify fut.arm = fut.arm.simple in the batss.glm function argument
futility1.arm.fun = function(posterior, b.fut){
  posterior < b.fut
}
```

```r

# specify RAR function
# Note that this function corresponds to the RAR.trippa function
# provided in BATSS, so instead you could specify RAR = RAR.trippa
# in the batss.glm function argument
prob.trippa = function(posterior, n, N, ref, active, gamma, eta, nu){
  g = sum(active) # (K_j+1 in the above)
  h = gamma*(sum(n)/N)^eta
  # vector of allocation probabilities:
  prob = rep(NA,g)
  # reference/control arm allocation - Equation 3:
  prob[1] = (exp(max(n[!ref])-n[ref])^nu)/(g-1)
  # targets/interventions (that haven't been dropped) - Equation 4:
  prob[2:g] = (posterior^h)/(sum(posterior^h))
  prob
}

#run the batss.glm function
sim_ex2.1 = batss.glm(model           = y~group,
                      var             = list(y = rbinom, group = group.fun),
                      var.control     = list(y = list(size = 1)),
                      family          = "binomial",
                      link            = "logit",
                      beta            = c(bb0, bb1, bb2, bb3, bb4, bb5),
                      which           = c(2:6),
                      alternative     = c("greater"),
                      R               = 10000,
                      N               = 216,
                      interim         = list(recruited=list(m0 = 60, m = 12)),
                      prob0           = c(A=1/6,B=1/6,C=1/6,D=1/6,E=1/6,F=1/6),
                      eff.arm         = efficacy.arm.fun,
                      delta.eff       = c(rep(NA,13), 0),
                      eff.arm.control = list(b.eff = 0.045),
                      delta.fut       = delta.f,
                      fut.arm         = futility1.arm.fun,
                      fut.arm.control = list(b.fut = 0.1),
                      RAR             = prob.trippa,
                      delta.RAR       = 0,
                      RAR.control     = list(gamma=3,
                                             eta=1.4,
                                             nu=1/10),
                      H0              = FALSE, # use TRUE to also simulate under H0
                      computation     = "parallel",
                      mc.cores        = 12,
                      extended        = 1)
```

1.3 Case Study 2.1 output

The extended output can be obtained using the following:

```
summary(sim_ex2.1)
```

This displays the following output:

```
---- Bayesian Adaptive Design with Laplace Approx. -----------------------------
     (10000 Monte Carlo samples)

-- Variables:
  * y : rbinom
  * group : alloc.balanced

-- Group randomisation:
  * prob.trippa

-- Model:
  * y ~ group (with logit link)

-- Fixed effect parameters:
            id target  Beta (H1)
 1 (Intercept)  FALSE -0.4054651
 2      groupB   TRUE  0.0000000
 3      groupC   TRUE  0.0000000
 4      groupD   TRUE  0.4054651
 5      groupE   TRUE  1.2527630
 6      groupF   TRUE  1.2527630

-- Sample size per interim analyis:
       id   n  m
  1  n=60  60 60
  2  n=72  72 12
  3  n=84  84 12
  4  n=96  96 12
  5 n=108 108 12
  6 n=120 120 12
  7 n=132 132 12
  8 n=144 144 12
  9 n=156 156 12
 10 n=168 168 12
 11 n=180 180 12
 12 n=192 192 12
 13 n=204 204 12
 14 n=216 216 12
```

```
---- H1: Under the alternative hypothesis ----

-- Target parameters:
             id alternative group efficacy futility
 1       groupB     greater     B   0.0449   0.5180
 2       groupC     greater     C   0.0409   0.5172
 3       groupD     greater     D   0.2113   0.2855
 4       groupE     greater     E   0.8977   0.0333
 5       groupF     greater     F   0.9003   0.0280
   At least one                     0.9819   0.7232
            All                     0.0063   0.0038

-- Efficacy:
             id alternative group early   last overall
 1       groupB     greater     B     0 0.0449  0.0449
 2       groupC     greater     C     0 0.0409  0.0409
 3       groupD     greater     D     0 0.2113  0.2113
 4       groupE     greater     E     0 0.8977  0.8977
 5       groupF     greater     F     0 0.9003  0.9003
   At least one                       0 0.9819  0.9819
            All                       0 0.0063  0.0063

-- Futility:
             id alternative group  early   last overall
 1       groupB     greater     B 0.5087 0.0093  0.5180
 2       groupC     greater     C 0.5083 0.0089  0.5172
 3       groupD     greater     D 0.2797 0.0058  0.2855
 4       groupE     greater     E 0.0333 0.0000  0.0333
 5       groupF     greater     F 0.0279 0.0001  0.0280
   At least one                   0.7129 0.0227  0.7232
            All                   0.0038 0.0000  0.0038

-- Sample size per group:
      id    ESS St.Dev q(0.1) q(0.5) q(0.9)
 1     A  49.89   9.49     41     48     62
 2     B  20.58   9.74     10     19     35
 3     C  20.34   9.67     10     18     35
 4     D  28.95  12.20     10     31     43
 5     E  47.71  10.65     39     48     59
 6     F  48.01  10.46     39     48     59
   Total 215.49   8.54    216    216    216

-- Scenarios:
        id groupB groupC groupD groupE groupF overall early
   1 00011      0      0      0      1      1  0.1322     0
   2 22011      2      2      0      1      1  0.1080     0
```

```
   3 22211      2      2      2      1      1  0.1037     0
   4 02011      0      2      0      1      1  0.0762     0
   5 20011      2      0      0      1      1  0.0754     0
   6 00111      0      0      1      1      1  0.0719     0
   7 20211      2      0      2      1      1  0.0334     0
   8 02211      0      2      2      1      1  0.0329     0
   9 20111      2      0      1      1      1  0.0294     0
  10 22111      2      2      1      1      1  0.0284     0
  .......
# omitted 112 rows here
 where 0 = no stop, 1 = efficacy stop, 2 = futility stop
```

1.4 Interpretation of Output (Case Study 2.1)

The first section of the output (up to the heading "`H1: Under the alternative hypothesis`") summarises the design and inputs into the `batss.glm` function.

The output under the heading "`Target parameters:`" provides a summary of how often (the proportion of the R simulated trials) each arm declared efficacy or futility (under the "efficacy" and "futility" headings, respectively), and how often at least one arm ("At least one" row), and how often all arms ("All" row) declared efficacy and futility. For example, arm B declared efficacy in 4.49% of the 10,000 simulated trials and futility in 51.8%. These efficacy or futility stops could occur at an interim analysis or at the maximum sample size (i.e., this is an overall summary of efficacy and futility stops, regardless of when they occurred).

The output under the heading "`Efficacy:`" provides a summary of how often each arm had an early stop (at an interim analysis) for efficacy ("early" column), and how often each arm had a declaration of efficacy at the maximum sample size (last look; "last" column), and the overall proportion of efficacy declarations (sum of the "early" and "last" columns; same as the "efficacy" column in the previous output table). The last two rows also provide how often at least one arm ("At least one" row) and how often all arms ("All" row) declared efficacy early and at the last look. Here we do not permit early stopping for efficacy, so the "early" column has 0s.

Similarly, the output under the heading "`Futility:`" summarises how often each arm, any arm and all arms had an early stop for futility, declared futility at the last look, and overall how often futility was declared. For example, arm B stopped early for futility in 50.83% of the trials, and declared futility in 0.9% of the trials at the maximum sample size (last look).

The "`Sample size per group:`" section summarises the expected sample size ("ESS"; mean sample size), the standard deviation of the sample sizes ("St.Dev") and the 10th, 50th and 90th percentiles of the sample sizes for each arm, and overall.

For example, arm A had a mean sample size of 49.89 with a standard deviation (SD) of 9.49. The overall mean (SD) sample size was 215.51 (8.54) patients.

Finally, the "`Scenarios:`" section provides a table listing the different combinations of conclusions that were reached for each intervention arm and what proportion of the simulated trials had that combination of conclusions ("overall" column; and of those proportions, what proportion were early stops on all arms - "early" column). The "group" columns ("groupB", ..., "groupF") list the conclusions for each arm: 0 = neither efficacy or futility criteria were met ("no stop"), 1 = efficacy criterion was met ("efficacy stop"), 2 = futility criterion was met ("futility stop') - at any look. If a "3" is displayed then both the efficacy and futility criteria were met for that arm. For example, in the first row we can see that arms B, C, and D did not reach a conclusion and arms E and F declared efficacy, with 13.22% of the simulations having this particular combination of conclusions (or lack of conclusions) for these arms.

We can also obtain plots summarising the output using the following commands:

```
# to plot the target parameter estimates vs sample size
# at the time of arm stopping for each of the R simulated trials:
plot(sim_ex2.1, which = 4)
# where cex and ylim options can also be used

# to plot the sample size per group across the R simulated trials:
plot(sim_ex2.1, which = 2)
```

If the user has selected to use `extended=1` or `extended=2` in the `batss.glm` call, then they can also view additional output that is saved to the `BATSS` object, such as the posterior estimates of the treatment effect at the final look for each arm and the results of each interim analysis for a particular Monte Carlo trial, using, for example:

```
# output for the first simulated trial of the 1...R simulated trials:
sim_ex2.1$H1$trial[1]
```

which displays the following output

```
[[1]]
[[1]]$target
       pos     id alternative group active look efficacy futility          low
groupB   1 groupB     greater     B  FALSE    6       NA     TRUE -1.48210875
groupC   2 groupC     greater     C  FALSE    1       NA     TRUE -3.22393923
groupD   3 groupD     greater     D  FALSE    1       NA     TRUE -3.22393916
groupE   4 groupE     greater     E  FALSE   14     TRUE       NA  0.03353878
groupF   5 groupF     greater     F  FALSE    1       NA     TRUE -3.22393923
              mid      high
groupB -0.4030409 0.6760269
groupC -1.3741886 0.4755621
groupD -1.3741886 0.4755620
```

```
groupE  0.6594757 1.2854126
groupF -1.3741886 0.4755621

[[1]]$look
      pos    id   n  m n(A) n(B) n(C) n(D) n(E) n(F) pe(groupB) pe(groupC)
n=60    1  n=60  60 60   10   10   10   10   10   10         NA         NA
n=72    2  n=72  72 12   15   13   10   10   14   10         NA         NA
n=84    3  n=84  84 12   18   18   10   10   18   10         NA         NA
n=96    4  n=96  96 12   23   21   10   10   22   10         NA         NA
n=108   5 n=108 108 12   26   23   10   10   29   10         NA         NA
n=120   6 n=120 120 12   32   24   10   10   34   10         NA         NA
n=132   7 n=132 132 12   38   24   10   10   40   10         NA         NA
n=144   8 n=144 144 12   45   24   10   10   45   10         NA         NA
n=156   9 n=156 156 12   51   24   10   10   51   10         NA         NA
n=168  10 n=168 168 12   57   24   10   10   57   10         NA         NA
n=180  11 n=180 180 12   63   24   10   10   63   10         NA         NA
n=192  12 n=192 192 12   69   24   10   10   69   10         NA         NA
n=204  13 n=204 204 12   75   24   10   10   75   10         NA         NA
n=216  14 n=216 216 12   81   24   10   10   81   10         NA         NA
      pe(groupD) pe(groupE) pe(groupF) pf(groupB) pf(groupC) pf(groupD)
n=60          NA         NA         NA 0.17143375 0.02949075 0.02949074
n=72          NA         NA         NA 0.47717837         NA         NA
n=84          NA         NA         NA 0.27078690         NA         NA
n=96          NA         NA         NA 0.15467878         NA         NA
n=108         NA         NA         NA 0.11142162         NA         NA
n=120         NA         NA         NA 0.07064381         NA         NA
n=132         NA         NA         NA         NA         NA         NA
n=144         NA         NA         NA         NA         NA         NA
n=156         NA         NA         NA         NA         NA         NA
n=168         NA         NA         NA         NA         NA         NA
n=180         NA         NA         NA         NA         NA         NA
n=192         NA         NA         NA         NA         NA         NA
n=204         NA         NA         NA         NA         NA         NA
n=216         NA  0.9804844         NA         NA         NA         NA
      pf(groupE) pf(groupF)      r(A)      r(B) r(C) r(D)      r(E) r(F)
n=60   0.1714337 0.02949075 0.3333333 0.3333333   NA   NA 0.3333333   NA
n=72   0.7111188         NA 0.3114933 0.3185841   NA   NA 0.3699226   NA
n=84   0.6823457         NA 0.3333333 0.2624990   NA   NA 0.4041677   NA
n=96   0.7820335         NA 0.3114933 0.1991699   NA   NA 0.4893368   NA
n=108  0.6840582         NA 0.4029599 0.1351606   NA   NA 0.4618795   NA
n=120  0.6673901         NA 0.5498340        NA   NA   NA 0.4501660   NA
n=132  0.6011325         NA 0.5498340        NA   NA   NA 0.4501660   NA
n=144  0.5544457         NA 0.5000000        NA   NA   NA 0.5000000   NA
n=156  0.3381709         NA 0.5000000        NA   NA   NA 0.5000000   NA
n=168  0.3754971         NA 0.5000000        NA   NA   NA 0.5000000   NA
n=180  0.5568735         NA 0.5000000        NA   NA   NA 0.5000000   NA
```

```
n=192  0.6474256         NA 0.5000000        NA   NA   NA 0.5000000   NA
n=204  0.7777777         NA 0.5000000        NA   NA   NA 0.5000000   NA
n=216  0.7862358         NA        NA        NA   NA   NA        NA   NA
[[1]]$data
NULL
```

For each simulated trial, this output provides the following information.

The first element of the list, named `$target`, indicates, for each target parameter (rows):

- `id`: the target parameter ID,
- `alternative`: the direction of the one-sided test for the target parameter of interest,
- `group`: the corresponding treatment group (useful when interaction terms are involved),
- `active`: whether the target parameter was still active at the last look (`FALSE` if the corresponding treatment group was stopped early, `TRUE` otherwise),
- `look`: the last look at which the target parameter was estimated,
- `efficacy` and `futility`: whether efficacy or futility was declared for the parameter when last evaluated,
- `low`, `mid`, `high`: the mean (`mid`) and 2.5% and 97.5% quantiles (`low`, `high`) from the posterior distribution of the target parameter when last evaluated.

The second element of the list, named `$look`, indicates, for each look (rows):

- `n`: the cumulative sample size at the given look,
- `m`: the number of participants recruited since the previous look,
- `n(A), n(B), n(C), …`: the cumulative sample size per group at the given look,
- `pe(groupB), pe(groupC), …`: the posterior probability of exceeding the user-specified clinically important difference for efficacy, `delta.eff`, i.e., $\Pr(\beta_k < \delta_e \mid \mathbf{y}, \mathbf{X})$ or $\Pr(\beta_k > \delta_e \mid \mathbf{y}, \mathbf{X})$, depending on the direction of the alternative hypothesis,
- `pf(groupB), pf(groupC), …`: the posterior probability of exceeding the user-specified clinically important difference for futility, `delta.fut`, i.e., $\Pr(\beta_k < \delta_f \mid \mathbf{y}, \mathbf{X})$ or $\Pr(\beta_k > \delta_f \mid \mathbf{y}, \mathbf{X})$, depending on the direction of the alternative hypothesis. Note that `pe` and `pf` coincide when `delta.eff` = `delta.fut`,

- `r(control)`, `r(A)`, `r(B)`, …: the allocation probabilities for each group, used to randomise the next `m` participants (`NA` for inactive arms).

The third element of the list, named `$data` (only available when `extended=2`), contains the simulated dataset for the given trial, including the look at which each participant was recruited, their outcome value, treatment group, and any other predictor values.

For illustration, in trial 1 (generated here with seed 1), groups C, D, and F were all stopped for futility at the first interim analysis, as their posterior probabilities of exceeding δ_f (i.e., `pf(·)`) fell below `b.fut` $= 0.1$ (`pf(C)` $=$ `pf(D)` $=$ `pf(F)` ≈ 0.029). Group B survived the first interim (`pf(B)` $= 0.171$) but was subsequently stopped for futility at look 6 (`pf(B)` $= 0.071 < 0.1$). Only group E remained active throughout the trial and was declared efficacious at the final look (look 14), with `pe(E)` $= 0.98$ exceeding the efficacy threshold $1-$ `b.eff` $= 1 - 0.045 = 0.955$. The effect of RAR is visible in the allocation probabilities: after groups C, D, and F were dropped at look 1, allocation among the three remaining groups (A, B, E) progressively shifted toward group E (from equal allocation at look 1 to $r(A) = 0.403$, $r(B) = 0.135$, $r(E) = 0.462$ by look 5). Once group B was also dropped at look 6, allocation was split equally between the control (A) and group E for the remainder of the trial.

1.5 Fixed-Design Code (Case Study 2.1)

Here we provide the R code and output for Case Study 2.1 when using a fixed design for the "Alternative" scenario and considering the same efficacy and futility parameter values for `b.eff` and `b.fut` as well as the same maximum sample size of $N = 216$ participants. The way to fit a fixed design with `BATSS` is simply to set `interim` to `NA`. The `RAR`-related arguments can be ignored as they have no effect in a design with a single analysis.

```

library(BATSS)

sim_ex2.1_fixed = batss.glm(model = y~group,
               var             = list(y = rbinom, group = group.fun),
               var.control     = list(y = list(size = 1)),
               family          = "binomial",
               link            = "logit",
               beta            = c(bb0, bb1, bb2, bb3, bb4, bb5),
               which           = c(2:6),
               alternative     = c("greater"),
               R               = 10000,
               N               = 216,
```

```
                   interim              = NA,
                   prob0                = c(A=1/6,B=1/6,C=1/6,D=1/6,E=1/6,F=1/6),
                   eff.arm              = efficacy.arm.fun,
                   delta.eff            = 0,
                   eff.arm.control      = list(b.eff = 0.045),
                   delta.fut            = delta.f,
                   fut.arm              = futility1.arm.fun,
                   fut.arm.control      = list(b.fut = 0.1),
                   H0                   = FALSE, # use TRUE to also simulate under H0
                   computation          = "parallel",
                   mc.cores             = 12,
                   extended             = 1)
```

Table ?? shows selected operating characteristics of the fixed design. Compared to those obtained with the adaptive design described in Section 4 (Table 3 of the manuscript), we can note that, when using the same efficacy and futility parameter values:

- the pairwise type I error rates and the family-wise error rate increase very slightly,
- the power decreases from approximately 90% to 82.5% for Arms E and F (the two effective arms),
- as expected, the probability of early stopping for futility and the sample size standard deviations equal zero, as all simulated trials recruit to the maximum sample size of $N = 216$.

This illustrates the potential benefits of the adaptive design, which in this setting achieves higher power by dropping futile arms early and reallocating patients to more promising treatments via RAR, while maintaining comparable type I error control.

2 Case Study 2.2 - "Alternative" scenario

Here we provide the R code and output for Case Study 2.2 from the main manuscript (with an increasing cut-off for the futility stopping boundary) for the "Alternative" scenario.

2.1 Case Study 2.2 Code

```

library(BATSS)

# assumed true ORR for each arm:
```

Group	Conclusion		Sample size	
	Efficacy(1)	Futility(2)	Average(3)	Stand. Dev.(4)
**Under the global null hypothesis:**				
Control (Arm A)	-	-	36	0
Arm B	0.052	0	36	0
Arm C	0.051	0	36	0
Arm D	0.051	0	36	0
Arm E	0.055	0	36	0
Arm F	0.052	0	36	0
At least 1	0.166	0	-	-
All	0.002	0	216	0
**Under the alternative hypothesis:**				
Control (Arm A)	-	-	36	0
Arm B	0.052	0	36	0
Arm C	0.051	0	36	0
Arm D	0.208	0	36	0
Arm E	0.819	0	36	0
Arm F	0.826	0	36	0
At least 1	0.933	0	-	-
All	0.011	0	216	0

Table C.1: (1) Probability of concluding a treatment arm, at least one treatment arm, or all treatment arms (arms B to F) to be effective at the end of the trial, (2) probability of stopping early a treatment arm, at least one treatment arm, or all treatment arms for futility (arms B to F), (3)(4) expected sample size per arm (A to F) and in total, and corresponding standard deviation, under the null and alternative hypotheses (refer to Table 2) in Case study 2.1

```
p0=.40; p1=.40; p2=.40; p3=.50; p4=.70; p5=.70
# beta parameters on log-OR scale:
bb0=log(p0/(1-p0)) #control arm / intercept
bb1=log((p1/(1-p1))/(p0/(1-p0))) #arm B tr effect
bb2=log((p2/(1-p2))/(p0/(1-p0)))
bb3=log((p3/(1-p3))/(p0/(1-p0)))
bb4=log((p4/(1-p4))/(p0/(1-p0)))
bb5=log((p5/(1-p5))/(p0/(1-p0))) #arm F tr effect

# calculation of delta.futility for a 10% absolute improvement over control
pp=p0+0.1
delta.f=log((pp/(1-pp))/(p0/(1-p0)))

# specify group allocation function. Note that we use this function,
# rather than the one specified in the main manuscript,
# as it leads to allocations that more closely match the intended randomisation
# probabilities. Note also that this function corresponds to
# the function alloc.balanced available in the BATSS package
group.fun= function(m, prob){
  prob = abs(prob)/sum(abs(prob)) #normalise probabilities
  m0.g = floor(prob*m)
  m0   = sum(m0.g)
```

```
  factor(rep(names(prob),m0.g+rmultinom(1,m-m0,prob)),
         levels=names(prob))
}


# specify function to declare efficacy for an arm
# stopping for efficacy only occurs at the last look (maximum sample size)
efficacy.arm.fun = function(posterior, b.eff){
   posterior > 1-b.eff
}
# where delta.eff needs to have NAs to not evaluate efficacy at interims

# specify function to declare futility for an arm
futility2.arm.fun = function(posterior, n, N,b.fut, p.fut){
  posterior < b.fut*(sum(n)/N)^p.fut
}


# specify RAR function.  Note that this function corresponds to
# the RAR.trippa function provided in BATSS, so that instead
# you could specify RAR = RAR.trippa in the batss.glm function argument
prob.trippa = function(posterior, n, N, ref, active, gamma, eta, nu){
  g = sum(active) # (K_j+1 in the above)
  h = gamma*(sum(n)/N)^eta
  # vector of allocation probabilities:
  prob = rep(NA,g)
  # reference/control arm allocation - Equation 3:
  prob[1] = (exp(max(n[!ref])-n[ref])^nu)/(g-1)
  # targets/interventions (that haven't been dropped) - Equation 4:
  prob[2:g] = (posterior^h)/(sum(posterior^h))
  prob
}


#run the batss.glm function
sim_ex2.2  = batss.glm(model        = y~group,
               var                  = list(y = rbinom, group = group.fun),
               var.control          = list(y = list(size = 1)),
               family               = "binomial",
               link                 = "logit",
               beta                 = c(bb0, bb1, bb2, bb3, bb4, bb5),
               which                = c(2:6),
               alternative          = c("greater"),
               R                    = 10000,
               N                    = 216,
               interim              = list(recruited=list(m0 = 60, m = 12)),
```

```
                prob0              = c(A=1/6,B=1/6,C=1/6,D=1/6,E=1/6,F=1/6),
                eff.arm            = efficacy.arm.fun,
                delta.eff          = c(rep(NA,13), 0),
                eff.arm.control    = list(b.eff = 0.045),
                delta.fut          = delta.f,
                fut.arm            = futility2.arm.fun,
                fut.arm.control    = list(b.fut = 0.8, p.fut = 1.8),
                RAR                = prob.trippa,
                delta.RAR          = 0,
                RAR.control        = list(gamma=3,
                                          eta=1.4,
                                          nu=1/10),
                H0                 = FALSE, # use TRUE to also simulate under H0
                computation        = "parallel",
                mc.cores           = 12,
                extended           = 1)
```

2.2 Case Study 2.2 output

The (extended) output can be obtained using the following command:

```
summary(sim_ex2.2)
```

The output from the summary function is provided below.

```
---- Bayesian Adaptive Design with Laplace Approx.------------------------------
     (10000 Monte Carlo samples)

-- Variables:
  * y : rbinom
  * group : alloc.balanced

-- Group randomisation:
  * prob.trippa

-- Model:
  * y ~ group (with logit link)

-- Fixed effect parameters:
            id target  Beta (H1)
 1 (Intercept)  FALSE -0.4054651
 2      groupB   TRUE  0.0000000
 3      groupC   TRUE  0.0000000
 4      groupD   TRUE  0.4054651
 5      groupE   TRUE  1.2527630
 6      groupF   TRUE  1.2527630

```

```
-- Sample size per interim analyis:
       id   n  m
  1  n=60  60 60
  2  n=72  72 12
  3  n=84  84 12
  4  n=96  96 12
  5 n=108 108 12
  6 n=120 120 12
  7 n=132 132 12
  8 n=144 144 12
  9 n=156 156 12
 10 n=168 168 12
 11 n=180 180 12
 12 n=192 192 12
 13 n=204 204 12
 14 n=216 216 12


---- H1: Under the alternative hypothesis ----

-- Target parameters:
             id alternative group efficacy futility   both
 1       groupB     greater     B   0.0406   0.9566 0.0013
 2       groupC     greater     C   0.0376   0.9597 0.0010
 3       groupD     greater     D   0.1873   0.7996 0.0119
 4       groupE     greater     E   0.8625   0.1266 0.0109
 5       groupF     greater     F   0.8728   0.1183 0.0088
   At least one                     0.9675   0.9925 0.0335
            All                     0.0057   0.0275 0.0000

-- Efficacy:
             id alternative group early   last overall
 1       groupB     greater     B     0 0.0406  0.0406
 2       groupC     greater     C     0 0.0376  0.0376
 3       groupD     greater     D     0 0.1873  0.1873
 4       groupE     greater     E     0 0.8625  0.8625
 5       groupF     greater     F     0 0.8728  0.8728
   At least one                       0 0.9675  0.9675
            All                       0 0.0057  0.0057

-- Futility:
             id alternative group  early   last overall
 1       groupB     greater     B 0.9313 0.0253  0.9566
 2       groupC     greater     C 0.9346 0.0251  0.9597
 3       groupD     greater     D 0.7481 0.0515  0.7996
 4       groupE     greater     E 0.1154 0.0112  0.1266
```

```
 5        groupF     greater     F 0.1056 0.0127  0.1183
   At least one                  0.9852 0.1146  0.9925
            All                  0.0248 0.0000  0.0275

-- Sample size per group:
      id    ESS St.Dev q(0.1) q(0.5) q(0.9)
 1     A  53.86  10.09     43     53     65
 2     B  18.66   8.65     10     16     32
 3     C  18.48   8.55     10     16     32
 4     D  26.23  12.01     10     25     42
 5     E  48.33  12.13     38     49     61
 6     F  48.82  11.86     38     49     61
   Total 214.38  12.52    216    216    216

-- Scenarios:
        id groupB groupC groupD groupE groupF overall early
  1 22211      2      2      2      1      1  0.5533   0.0
  2 22111      2      2      1      1      1  0.1344   0.0
  3 22221      2      2      2      2      1  0.0876   0.0
  4 22212      2      2      2      1      2  0.0799   0.0
  5 22222      2      2      2      2      2  0.0275   0.9
  6 12111      1      2      1      1      1  0.0157   0.0
  7 21111      2      1      1      1      1  0.0145   0.0
  8 12211      1      2      2      1      1  0.0136   0.0
  9 21211      2      1      2      1      1  0.0129   0.0
 10 22311      2      2      3      1      1  0.0093   0.0
 11 22121      2      2      1      2      1  0.0069   0.0
 12 22231      2      2      2      3      1  0.0069   0.0
 13 22213      2      2      2      1      3  0.0066   0.0
 14 11111      1      1      1      1      1  0.0057   0.0
 15 22112      2      2      1      1      2  0.0053   0.0
 16 22232      2      2      2      3      2  0.0028   0.0
 17 11211      1      1      2      1      1  0.0022   0.0
 18 22223      2      2      2      2      3  0.0016   0.0
 19 22321      2      2      3      2      1  0.0013   0.0
 20 32211      3      2      2      1      1  0.0012   0.0
 .......
# omitted 38 rows here
where 0 = no stop, 1 = efficacy stop, 2 = futility stop,
      3 = simultaneous efficacy and futility stops
```

Plots can be obtained using the following command:

```
plot(sim_ex2.2, which = 1:4)
```

The following figures are then produced:

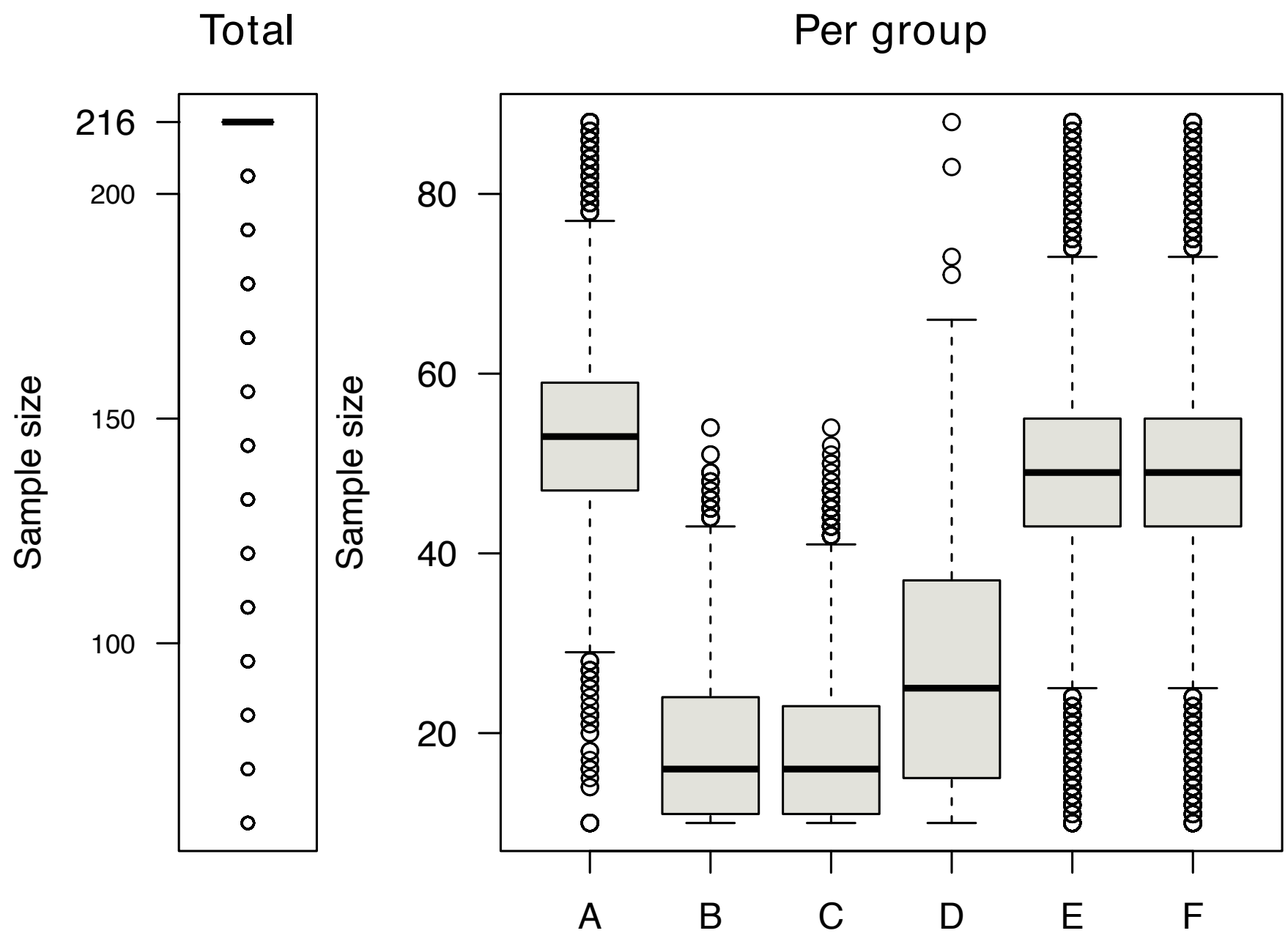

Figure C.1: Boxplots of the total sample size (left) and the sample size per group (right) as observed in the 10,000 simulated trials.

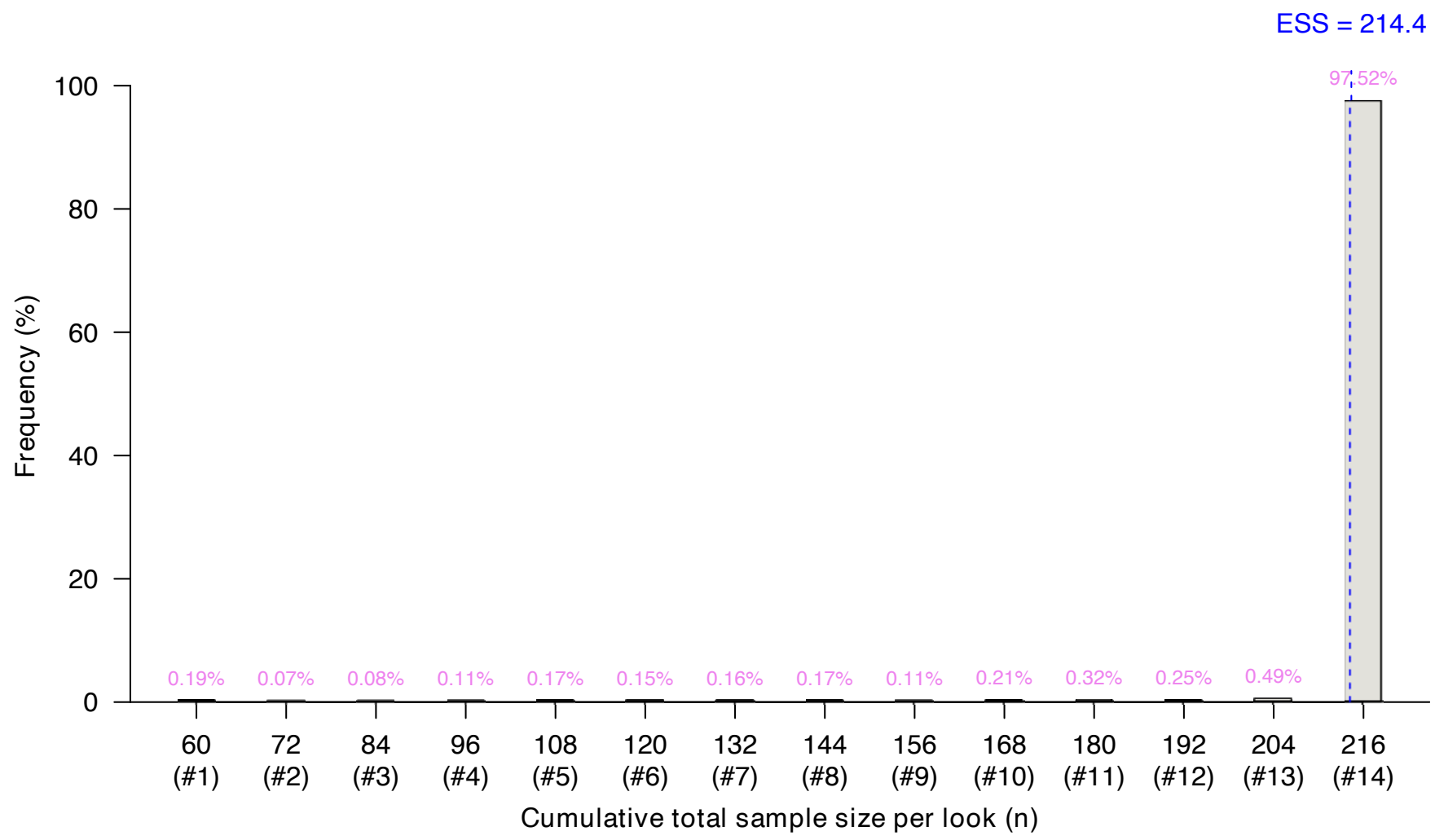

Figure C.2: Bar plot of the probability of stopping (y-axis) at each look (x-axis) as observed in the simulated samples. The vertical blue dashed line shows the estimate of the expected sample size (ESS).

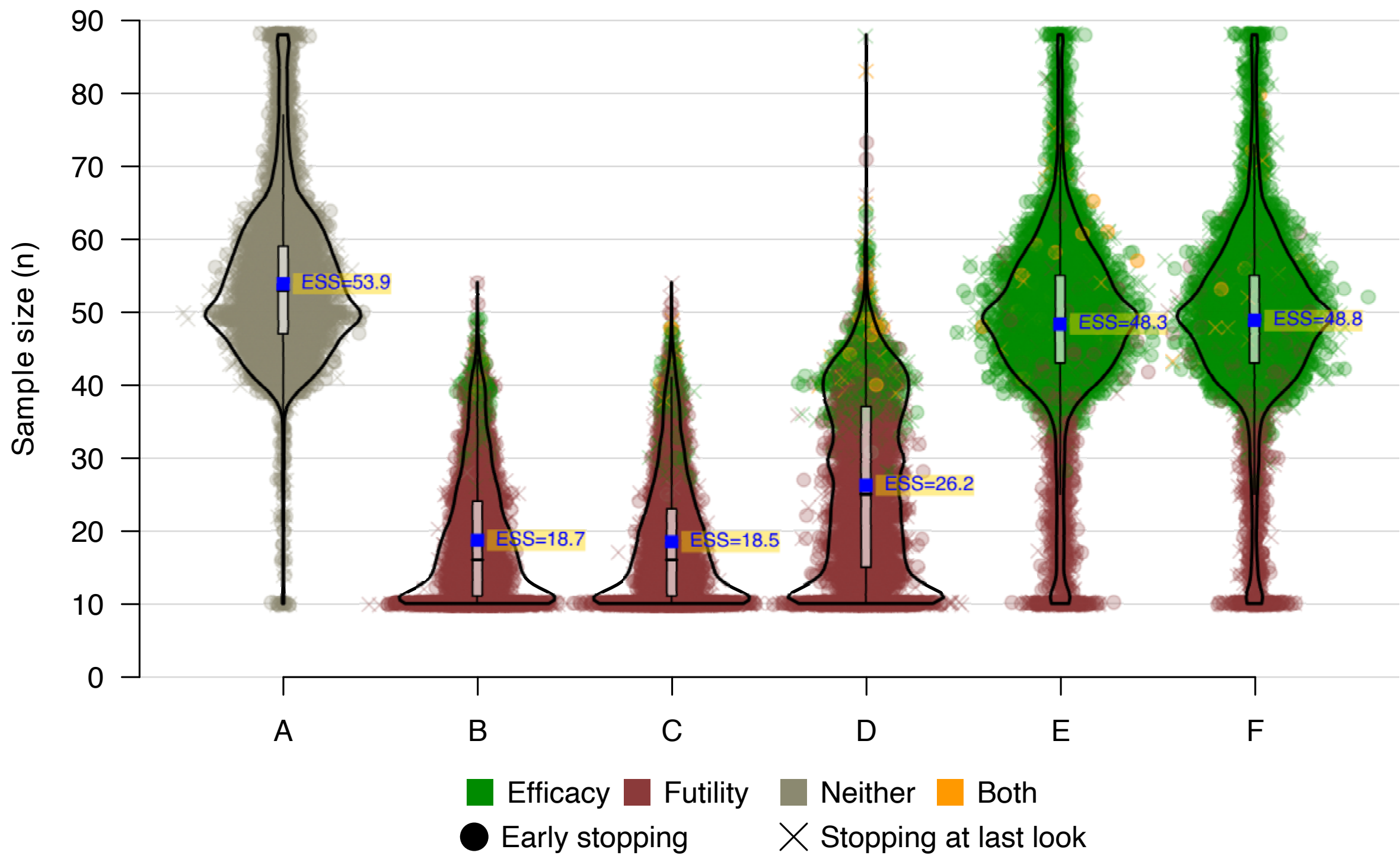

Figure C.3: Violin plot (and boxplot) of the simulated sample sizes per treatment arm for each trial and corresponding expected sample sizes. For target parameters, these points are colour-coded and shaped by the decision and timing when the arm stopped.

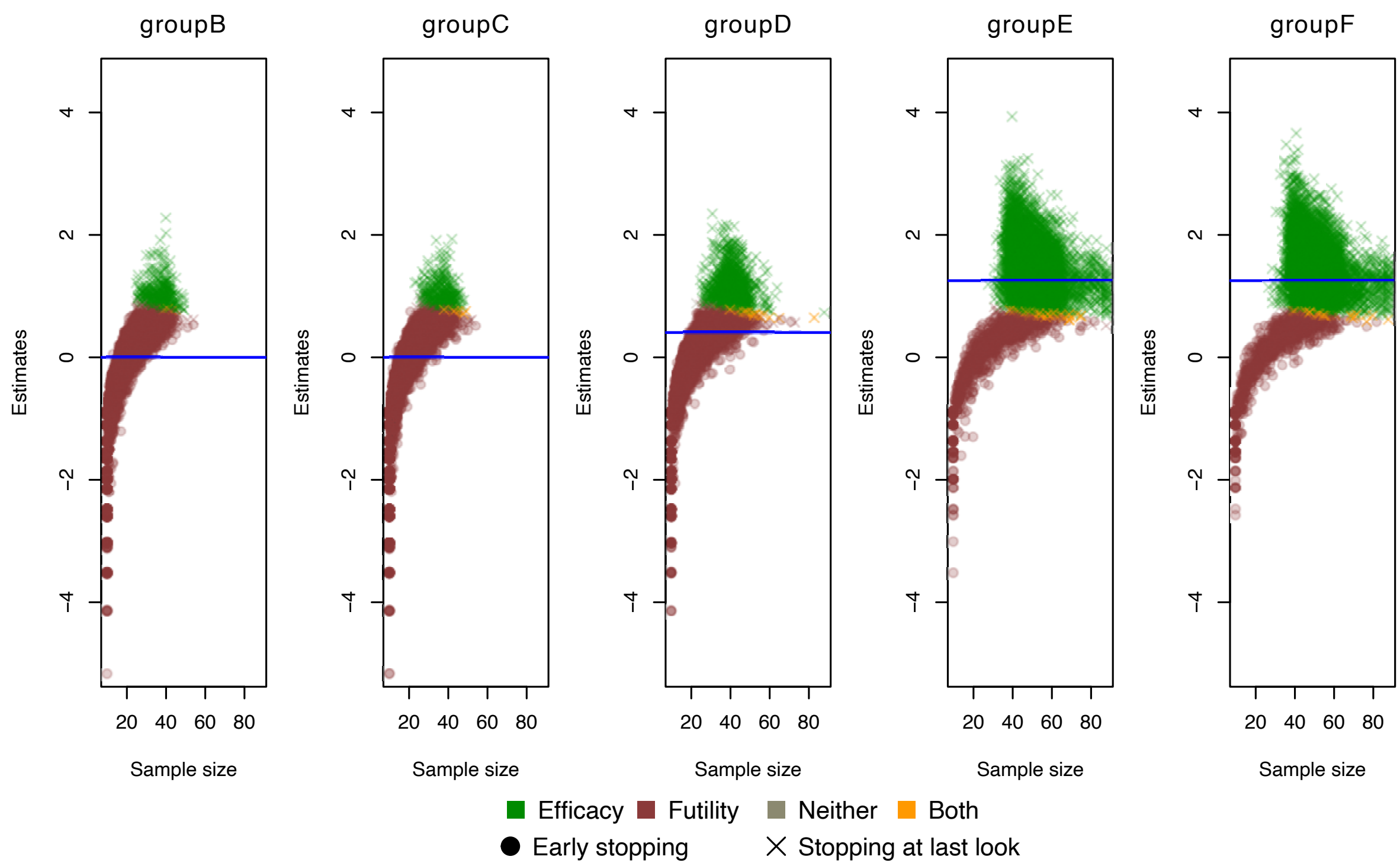

Figure C.4: Scatterplot of target parameter estimates (posterior mean) versus sample size for each intervention arm (separate plots) at the time of stopping the arm, along with the decision at the time of stopping for the simulated scenario for Case Study 2.2 across the 10,000 simulated trials. The timing of the stop is also provided ("Early stopping" = stopping at an interim analysis).The horizontal blue lines show the assumed target parameter values.

2.3 Interpretation of Outputs (Case Study 2.2)

It is important to note here that for some simulated trials, both the efficacy and futility criteria were simultaneously met. This is displayed under the "`Target parameters:`" section under the column "both" in the output provided by the `summary()` function. For example, we can see that arm D met both the efficacy and futility criteria in 1.19% of the simulated trials, and arm E met both criteria in 1.09% of the simulated trials.

The "`Scenarios:`" section of the output also displays a "3" for treatment arms that met both efficacy and futility criteria at the same look. For example, in row 10 of the Scenarios output, arm D met both criteria, whilst arms B and C met the futility criteria and arms E and F met the efficacy criteria; this combination of conclusions occurred in 0.84% of the simulated trials.

This behaviour is also displayed in Figure **??** ("Both" labelled in orange).

2.4 Fixed-Design Code (Case Study 2.2)

Here we provide the R code and output for Case Study 2.2 when using a fixed design for the "Alternative" scenario and considering the same efficacy and futility parameter values for `b.eff`, `b.fut` and `p.fut`, as well as the same maximum sample size of $N = 216$ participants. The way to fit a fixed design with `BATSS` is simply to set `interim` to `NA`. The `RAR`-related arguments can be ignored as they have no effect in a design with a single analysis.

```

library(BATSS)

#run the batss.glm function
#run the batss.glm function
sim_ex2.2_fixed = batss.glm(model = y~group,
               var              = list(y = rbinom, group = group.fun),
               var.control      = list(y = list(size = 1)),
               family           = "binomial",
               link             = "logit",
               beta             = c(bb0, bb1, bb2, bb3, bb4, bb5),
               which            = c(2:6),
               alternative      = c("greater"),
               R                = 10000,
               N                = 216,
               interim          = NA,
               prob0            = c(A=1/6,B=1/6,C=1/6,D=1/6,E=1/6,F=1/6),
               eff.arm          = efficacy.arm.fun,
               delta.eff        = 0,
               eff.arm.control  = list(b.eff = 0.045),
```

```
                delta.fut           = delta.f,
                fut.arm             = futility1.arm.fun,
                fut.arm.control     = list(b.fut = 0.8, p.fut = 1.8),
                H0                  = FALSE, # use TRUE to also simulate under H0
                computation         = "parallel",
                mc.cores            = 12,
                extended            = 1)
```

As the difference between the designs of Case Study 2.1 and Case Study 2.2 can only affect the assessment of futility at the final analysis, the selected operating characteristics of the fixed design are the same as in Table ??. Compared to those obtained with the adaptive design described in Section 4 (Table 4 of the manuscript), we note about the same differences as described in Section 1.5 of this appendix.

3 Run times

The run times for Case study 2 are displayed in Table ??

**Case**	**Scenario**	**Computer**	**Run time**
Case Study 2.1	Null	Cluster (500 CPUs)	8.0 minutes
Case Study 2.1	Alternative	Cluster (500 CPUs)	8.8 minutes
Case Study 2.2	Null	Cluster (500 CPUs)	6.3 minutes
Case Study 2.2	Alternative	Cluster (500 CPUs)	8.4 minutes
Case Study 2.1	Null	Standard Windows desktop (12 cores)	4.20 hours
Case Study 2.1	Alternative	Standard Windows desktop (12 cores)	4.74 hours
Case Study 2.2	Null	Standard Windows desktop (12 cores)	3.00 hours
Case Study 2.2	Alternative	Standard Windows desktop (12 cores)	4.78 hours

Table C.2: Run times for Case study 2 (with different futility stopping rules; Case Studies 2.1 and 2.2) for each scenario using 2 different computing set-ups.
