# Supplementary material for: A Fast, Flexible Simulation Framework for Bayesian Adaptive Designs—The R Package BATSS
Source: Pharm Stat. 2026 Aug 10;25(5):e70109. doi: 10.1002/pst.70109 (PMC13456979; doi:10.1002/pst.70109)
Supplement: Supplementary file 2 [file PST-25-0-s003.pdf]

Appendix B
Overdispersed count outcome case study
Full description, code and output

July 7, 2026

1 Case Study 1 Description

This case study is inspired by the CHANGE-MS study (Curtin et al., 2016; Hartung et al., 2022), a phase IIb randomised controlled trial that investigated the efficacy and safety of temelimab (GNbAC1), a monoclonal antibody targeting the pHERV-W envelope protein, for the treatment of relapsing-remitting multiple sclerosis (MS). The trial compared three doses of temelimab (6, 12, and 18 mg/kg, referred to here as arms "A", "B" and "C") against placebo (control), administered as monthly intravenous infusions over 48 weeks.

The primary endpoint was the cumulative number of new gadolinium-enhancing T1 lesions on brain MRI scans performed on four occasions between weeks 12 and 24 post-randomisation.

The data were overdispersed and modelled using a negative binomial distribution. The aim of the trial was to determine whether any of the three doses of temelimab reduced the number of active lesions compared to placebo, via pairwise comparisons of each dose to control. Refer to Section 3.1 of the main manuscript for a description of the design parameters used in our simulation.

Our case study has thus 4 treatment arms: placebo (control), low, medium and high doses of temelimab (intervention arms referred to as arms "A", "B" and "C", respectively). This is a superiority trial where we are interested in demonstrating efficacy compared to the placebo arm and intervention arms will not be compared to one another.

We will begin the interim analyses at 100 participants completing follow-up, and perform interim analyses every 40 participants completing follow-up thereafter. Our maximum sample size is 260 participants, which is the same as the original study

(Curtin et al., 2016). Similar to the original study, we aim to have 90% power to detect a 60% reduction (RR=0.4) in the mean cumulative number of lesions in the highest dose intervention arm (from a mean of 4 (control) to 1.6 lesions). A dispersion (shape) parameter with a value of 0.5 was assumed using the following parameterisation for the variance:

$$Var(Y) = \mu + \mu^2/\phi \tag{1}$$

(i.e., $\phi = 1/2$). The original study (Curtin et al., 2016; Hartung et al., 2022) did not have any interim analyses. Here we will use equal randomisation.

Early stopping of intervention arms for efficacy may occur if there is a high posterior probability of (any) benefit for intervention arm k ($k = 1, ..., K$ for intervention arms and $k = 0$ for control) at look j:

$$P(\beta_k < \delta_e | \ \mathbf{y}, \mathbf{X}) \quad > \quad 1 - b_e \cdot \left(\frac{\sum_{k=0}^{K} (n_{k,j})}{N} \right)^{p_e} \tag{2}$$

The idea behind this stopping rule is to use a slow-decreasing boundary as more information becomes available. This is akin to the frequentist setting where the upper boundary may become less stringent as the trial progresses. A reasonable proposal is 1 minus a power of a function of the fraction of information available in the trial so far. Here $\delta_e = 0$; $1 - b_e$ is the efficacy boundary at the maximum sample size; $\sum_{k=0}^{K}(n_{k,j})$ is the total number of participants enrolled at look j, and p_e is a tuning parameter that determines the shape of the function (Gotmaker et al., 2019). This can be written as an R function using:

```
efficacy.arm.fun = function(posterior, n, N, b.eff, p.eff){
    posterior > (1-(b.eff*(sum(n)/N)^p.eff))
}
```

We will stop an intervention arm for futility if there is a low posterior probability of seeing a minimum clinically important difference of a 20% reduction (i.e., RR=0.8):

$$P(\beta_k < \delta_f | \ \mathbf{y}, \mathbf{X}) < b_f$$

where $\delta_f = log(0.8)$ and $b_f = 0.2025$ (i.e., we used a fixed futility boundary value here). This can be written as an R function using:

```
futility.arm.fun = function(posterior, b.fut){
    posterior < b.fut
}
```

The trial will run until a decision has been reached for each intervention arm (stopping arms for efficacy or futility) or once the maximum sample size has been reached

(default option in `batss.glm`). That is, the trial will not stop early if just one arm has met the efficacy stopping criteria (although the user can specify stopping rules such that the trial stops as soon as a stopping rule has been met for one arm).

Here we aim to control the one-sided type I family-wise error at 5% (under the global null) and aim to have 90% power to detect a large treatment effect (RR=0.4). We performed grid searches to optimise the design parameters for the user-defined functions (p_e, b_e, b_f) so that suitable operating characteristics (namely type I error and power) were obtained. The δ values were chosen based on clinicians' input to incorporate clinically important differences.

We will explore 3 scenarios of interest when performing the simulations:

- "Global null", where the cumulative mean number of lesions = 4 for each arm
- "One treatment works" where there is a 60% reduction in arm C (high dose), i.e., RR=0.4, and arms A and B have the same cumulative mean number of lesions as the control (RR = 1)
- "Better, Best" where there is a 20% reduction in arm A (RR=0.8), 40% reduction in arm B (RR=0.6), and a 60% reduction in arm C (RR=0.4)

We simulated 10,000 trials under each scenario. The one-sided family-wise type I error was calculated as the proportion of simulations that falsely declared at least one of the interventions to be superior (demonstrate efficacy) to the control under the global null. The (pairwise) power was calculated as the proportion of simulations that declared efficacy for an intervention when the intervention had a large effect (i.e., RR=0.4).

2 Case Study 1 Code

The code to run the "One treatment works" scenario for Case Study 1 with both an adaptive and a fixed design is provided below.

2.1 Adaptive design

```
library(BATSS)

# specify group/treatment allocation function
# note that treatalloc.fun is the same as the alloc.balanced function
# that is provided in BATSS, so you could set
# treatment = alloc.balanced in the batss.glm function
# (in the `var' argument)
```

```
treatalloc.fun = function(m,prob){
  prob = abs(prob)/sum(abs(prob)) #normalise probabilities
  m0.g = floor(prob*m)
  m0   = sum(m0.g)
  factor(rep(names(prob),m0.g+rmultinom(1,m-m0,prob)),
         levels=names(prob))
}

# specify function to declare efficacy for an arm
# note that efficacy.arm.fun the same as the eff.arm.infofract function
# provided in BATSS, so you could set eff.arm = eff.arm.infofract
# in the batss.glm function
efficacy.arm.fun = function(posterior,n,N,b.eff,p.eff){
  posterior > (1-(b.eff*(sum(n)/N)^p.eff))
}

# specify function to declare futility for an arm
# note that futility.arm.fun is the same as the fut.arm.simple function
# provided in BATSS, so you could set fut.arm = fut.arm.simple
# in the batss.glm function
futility.arm.fun = function(posterior,b.fut){
  posterior < b.fut
}


scenario_1works = batss.glm(
                 model              = y~treatment,
                 var                = list(y = rnbinom,
                                           treatment = treatalloc.fun),
                 var.control        = list(y = list(size = 1/2)), # shape
                 family             = "nbinomial",
                 link               = "log",
                 beta               = c(log(4),log(1),log(1),log(0.4)),
                 which              = c(2:4),
                 alternative        = c("less"),
                 R                  = 10000,
                 N                  = 260,
                 interim            = list(recruited=c(100,140,180,220)),
                 prob0              = c(control = 1, A = 1, B = 1, C = 1),
                 eff.arm            = efficacy.arm.fun,
                 delta.eff          = 0,
                 eff.arm.control    = list(b.eff=0.009,
                                           p.eff=3),
                 delta.fut          = log(0.8),
                 fut.arm            = futility.arm.fun,
                 fut.arm.control    = list(b.fut=0.2025),
```

```
                   RAR                  = NULL,
                   H0                   = FALSE,
                   computation          = "parallel",
                   mc.cores             = 12,
                   extended             = 1)
```

The "size" in the `var.control` argument is the dispersion parameter in R's `rnbinom` function (ϕ in Equation 1).

If the user has selected to use `extended=1` or `extended=2` in the `batss.glm` call, they can also view additional output saved in the `BATSS` object, such as the posterior estimates of the treatment effect at the final look for each arm and the results of each interim analysis for a particular simulated trial, using, for example:

```
# output for the first simulated trial of the 1...R simulated trials:
scenario_1works$H1$trial[1]
```

which displays the following output

```
[[1]]
[[1]]$target
           pos          id alternative group active look efficacy
treatmentA   1 treatmentA        less     A   TRUE    5       NA
treatmentB   2 treatmentB        less     B   TRUE    5       NA
treatmentC   3 treatmentC        less     C  FALSE    1     TRUE
           futility        low        mid        high
treatmentA       NA -0.8786894 -0.4237062  0.03128296
treatmentB       NA -0.5756593 -0.1295440  0.31656354
treatmentC       NA -2.5753860 -1.8000741 -1.02477781

[[1]]$look
      pos    id   n   m n(control) n(A) n(B) n(C) pe(treatmentA)
n=100   1 n=100 100 100         25   25   25   25      0.9957926
n=140   2 n=140 140  40         39   38   38   25      0.9860121
n=180   3 n=180 180  40         52   51   52   25      0.9570784
n=220   4 n=220 220  40         65   64   66   25      0.9890575
n=260   5 n=260 260  40         78   77   80   25      0.9663645
      pe(treatmentB) pe(treatmentC) pf(treatmentA) pf(treatmentB)
n=100      0.8974280              1      0.9799537      0.7415779
n=140      0.8581472             NA      0.9315434      0.6326392
n=180      0.6030061             NA      0.8237169      0.2917522
n=220      0.7536436             NA      0.9215548      0.4127065
n=260      0.7170608             NA      0.8078221      0.3404367
      pf(treatmentC) r(control)      r(A)      r(B) r(C)
n=100      0.9999596  0.3333333 0.3333333 0.3333333   NA
n=140             NA  0.3333333 0.3333333 0.3333333   NA
n=180             NA  0.3333333 0.3333333 0.3333333   NA
n=220             NA  0.3333333 0.3333333 0.3333333   NA
n=260             NA         NA        NA        NA   NA

[[1]]$data
NULL
```

For each simulated trial, this output provides the following information.

The first element of the list, named `$target`, indicates, for each target parameter (rows):

- `id`: the target parameter ID,
- `alternative`: the direction of the one-sided test for the target parameter of interest,
- `group`: the corresponding treatment group (useful when interaction terms are involved),
- `active`: whether the target parameter was still active at the last look (`FALSE` if the corresponding treatment group was stopped early, `TRUE` otherwise),
- `look`: the last look at which the target parameter was estimated,
- `efficacy` and `futility`: whether efficacy or futility was declared for the parameter when last evaluated,
- `low`, `mid`, `high`: the mean (`mid`) and 2.5% and 97.5% quantiles (`low`, `high`) from the posterior distribution of the target parameter when last evaluated.

The second element of the list, named `$look`, indicates, for each look (rows):

- `n`: the cumulative sample size at the given look,
- `m`: the number of participants recruited since the previous look,
- `n(control), n(A), n(B), …`: the cumulative sample size per group at the given look,
- `pe(treatmentA), pe(treatmentB), …`: the posterior probability of exceeding the user-specified clinically important difference for efficacy, `delta.eff`, i.e., $\Pr(\beta_k < \delta_e \mid \mathbf{y}, \mathbf{X})$ or $\Pr(\beta_k > \delta_e \mid \mathbf{y}, \mathbf{X})$, depending on the direction of the alternative hypothesis,
- `pf(treatmentA), pf(treatmentB), …`: the posterior probability of exceeding the user-specified clinically important difference for futility, `delta.fut`, i.e., $\Pr(\beta_k < \delta_f \mid \mathbf{y}, \mathbf{X})$ or $\Pr(\beta_k > \delta_f \mid \mathbf{y}, \mathbf{X})$, depending on the direction of the alternative hypothesis. Note that `pe` and `pf` coincide when `delta.eff` = `delta.fut`,
- `r(control), r(A), r(B), …`: the allocation probabilities for each group, used to randomise the next `m` participants (`NA` for inactive arms).

The third element of the list, named `$data` (only available when `extended=2`), contains the simulated dataset for the given trial, including the look at which each participant was recruited, their outcome value, treatment group, and any other predictor values.

For illustration, in trial 1 (generated here with seed 1), we can note that treatment C was dropped at the first interim analysis for efficacy, since `pe(treatmentC)` $= 1$ at look 1 exceeded the efficacy threshold $1 - 0.009 \times (100/260)^3 \approx 0.9995$; that is, the posterior probability $\Pr(\beta_C < \delta_e \mid \mathbf{y}, \mathbf{X})$ was large enough to declare treatment C efficacious. The remaining arms continued until look 5, at which point neither was found efficacious. No arm was stopped for futility at any stage, as $\Pr(\beta_k < \delta_f \mid \mathbf{y}, \mathbf{X})$, i.e., `pf(·)`, never fell below `b.fut` $= 0.2025$. As no RAR was used, the allocation probabilities remained fixed (equal allocation with 1/4 at the first interim analysis when all four arms were active, and 1/3 afterwards, since group C was dropped at look 1).

2.2 Fixed design

Here we provide the R code when using a fixed design and considering the same efficacy and futility parameter values for `b.eff`, `p.eff` and `b.fut`, as well as the same maximum sample size of $N = 260$ participants. The way to fit a fixed design with `BATSS` is simply to set `interim` to `NA`.

```
library(BATSS)

scenario_1works_fixed = batss.glm(
                 model              = y~treatment,
                 var                = list(y = rnbinom,
                                           treatment = treatalloc.fun),
                 var.control        = list(y = list(size = 1/2)), # shape
                 family             = "nbinomial",
                 link               = "log",
                 beta               = c(log(4),log(1),log(1),log(0.4)),
                 which              = c(2:4),
                 alternative        = c("less"),
                 R                  = 10000,
                 N                  = 260,
                 interim            = NA,
                 prob0              = c(control = 1, A = 1, B = 1, C = 1),
                 eff.arm            = efficacy.arm.fun,
                 delta.eff          = 0,
                 eff.arm.control    = list(b.eff=0.009,
                                           p.eff=3),
                 delta.fut          = log(0.8),
                 fut.arm            = futility.arm.fun,
                 fut.arm.control    = list(b.fut=0.2025),
                 RAR                = NULL,
                 H0                 = FALSE,
```

```
                  computation         = "parallel",
                  mc.cores            = 12,
                  extended            = 1)
```

The output for the “One treatment works” scenario for Case Study 1 is presented below, first for the adaptive design and then for the fixed design.

3 Outputs

The output for the “One treatment works” scenario for Case Study 1 is presented below, first for the adaptive design and then for the fixed design.

3.1 Adaptive design

We can obtain the operating characteristics for the above scenario (“One treatment works”) using

```
    summary(scenario_1works)
```

This displays the following output:

```
---- Bayesian Adaptive Design with Laplace Approx.--------------------------
     (10000 Monte Carlo samples)

-- Variables:
  * y : rnbinom
  * treatment : treatalloc.fun

-- Model:
  * y ~ treatment (with log link)

-- Fixed effect parameters:
            id target  Beta (H1)
 1 (Intercept)  FALSE  1.3862944
 2  treatmentA   TRUE  0.0000000
 3  treatmentB   TRUE  0.0000000
 4  treatmentC   TRUE -0.9162907

-- Sample size per interim analyis:
      id   n   m
 1 n=100 100 100
 2 n=140 140  40
 3 n=180 180  40
 4 n=220 220  40
 5 n=260 260  40

```

```
---- H1: Under the alternative hypothesis ----

-- Target parameters:
             id alternative group efficacy futility
 1   treatmentA        less     A   0.0182   0.7075
 2   treatmentB        less     B   0.0194   0.7136
 3   treatmentC        less     C   0.9471   0.0108
   At least one                     0.9475   0.8415
            All                     0.0016   0.0105

-- Efficacy:
             id alternative group  early   last overall
 1   treatmentA        less     A 0.0128 0.0054  0.0182
 2   treatmentB        less     B 0.0126 0.0068  0.0194
 3   treatmentC        less     C 0.8680 0.0791  0.9471
   At least one                   0.8685 0.0906  0.9475
            All                   0.0010 0.0000  0.0016

-- Futility:
             id alternative group  early   last overall
 1   treatmentA        less     A 0.6577 0.0498  0.7075
 2   treatmentB        less     B 0.6645 0.0491  0.7136
 3   treatmentC        less     C 0.0108 0.0000  0.0108
   At least one                   0.8009 0.0942  0.8415
            All                   0.0105 0.0000  0.0105

-- Sample size per group:
        id    ESS St.Dev q(0.1) q(0.5) q(0.9)
 1 control  74.33  17.34     45     76     98
 2       A  49.42  25.10     25     39     85
 3       B  49.12  25.01     25     38     84
 4       C  52.77  21.40     25     48     85
     Total 225.65  44.04    140    260    260

-- Scenarios:
     id treatmentA treatmentB treatmentC overall early
  1 221          2          2          1  0.5399  0.80
  2 001          0          0          1  0.1329  0.00
  3 021          0          2          1  0.1221  0.00
  4 201          2          0          1  0.1166  0.00
  5 220          2          2          0  0.0292  0.00
  6 011          0          1          1  0.0115  0.00
  7 222          2          2          2  0.0105  1.00
  8 101          1          0          1  0.0088  0.00
  9 121          1          2          1  0.0077  0.60
```

```
10 211          2          1          1  0.0060  0.55
11 200          2          0          0  0.0050  0.00
12 020          0          2          0  0.0041  0.00
13 000          0          0          0  0.0034  0.00
14 111          1          1          1  0.0016  0.62
15 010          0          1          0  0.0002  0.00
16 202          2          0          2  0.0002  0.00
17 022          0          2          2  0.0001  0.00
18 100          1          0          0  0.0001  0.00
19 210          2          1          0  0.0001  0.00
where 0 = no stop, 1 = efficacy stop, 2 = futility stop
...
```

Interpretation of Output

The first section of the output (up to the heading "`H1: Under the alternative hypothesis`") summarises the design and inputs into the `batss.glm` function.

The overall proportion of simulations that declared efficacy and futility (at any look) for each intervention arm are displayed under the "Target parameters" heading (rows "treatmentA" - "treatmentC"; columns "efficacy" and "futility"). For example, treatment arm A met the efficacy criteria in 1.82% of the simulated trials and met the futility criteria in 70.75% of the simulated trials. The final two rows of this table (named "At least one" and "All") provide the proportion of simulations where at least one arm met the efficacy criteria or the futility criteria, and the proportion of simulations that all arms met the efficacy or futility criteria, respectively. For example in 94.75% of the simulations the efficacy criteria were met by at least one intervention arm and in 0.16% of the simulations the efficacy criteria were met by all intervention arms.

We can examine the proportion of early and late (at the last look at the maximum sample size) stopping for each intervention arm by looking at the "early" and "last" columns under the "Efficacy" and "Futility" headings. For example, treatment arm C stopped early for efficacy in 86.80% of the simulations (when assuming a large effect), and declared efficacy at the last look (at the maximum sample size) in 7.91% of the simulations (thus, overall, declaring efficacy in 94.71% of the simulations). Treatment A (assumed to be ineffective) stopped early for futility in 65.77% of the simulations, and declared futility at the last look in 4.98% of the simulations (thus, overall, declaring futility in 70.75% of the simulations).

Summaries of the sample size per arm and overall are provided in the "Sample size per group" section of the output. For example, the control arm had an average sample size of 74.33 (SD = 17.34), with a median sample size of 76 (10th percentile 45, 90th percentile 98 patients).

The “Scenarios” section of the output provides a table listing the different combinations of conclusions that were reached for each intervention arm and what proportion of the simulated trials had that combination of conclusions (“overall” column; and of those proportions, what proportion were early stops on all arms - “early” column). The “treatment” columns (“treatmentA”, “treatmentB”, “treatmentC”) list the conclusions for each arm: 0 = neither efficacy or futility criteria were met (“no stop”), 1 = efficacy criterion was met (“efficacy stop”), 2 = futility criterion was met (“futility stop’) - at any look. If a “3” is displayed then both the efficacy and futility criteria were met for that arm. In the first row we can see that arms A and B stopped for futility and arm C stopped for efficacy with 53.99% of the simulations having this particular combination of conclusions for these arms. Of those simulated trials that had this particular combination of conclusions, 80% of the trials stopped early.

3.2 Fixed design

We can obtain the operating characteristics for the above scenario (“One treatment works”) using

```
summary(scenario_1works_fixed)
```

This displays the following output:

```
---- Bayesian Fixed Design with Laplace Approx. ---------------------------
  (10000 Monte Carlo samples)

-- Variables:
  * y : rnbinom
  * treatment : treatalloc.fun

-- Decision rules:
  * Efficacy:  efficacy.arm.fun
  * Futility:  futility.arm.fun

-- Model:
  * y ~ treatment (with log link)

-- Fixed effect parameters:
            id target  Beta (H1)
 1 (Intercept)  FALSE  1.3862944
 2  treatmentA   TRUE  0.0000000
 3  treatmentB   TRUE  0.0000000
 4  treatmentC   TRUE -0.9162907


```

```
-- Sample size per interim analyis:
      id   n   m
 1 n=260 260 260

---- H1: Under the alternative hypothesis ----

-- Target parameters:
             id alternative group efficacy futility
 1   treatmentA        less     A   0.0121   0.5031
 2   treatmentB        less     B   0.0123   0.5052
 3   treatmentC        less     C   0.8573   0.0003
 1 At least one                     0.8575   0.6705
 2          All                     0.0016   0.0003

-- Efficacy:
             id alternative group early   last overall
 1   treatmentA        less     A     0 0.0121  0.0121
 2   treatmentB        less     B     0 0.0123  0.0123
 3   treatmentC        less     C     0 0.8573  0.8573
   At least one                       0 0.8575  0.8575
            All                       0 0.0016  0.0016

-- Futility:
             id alternative group early   last overall
 1   treatmentA        less     A     0 0.5031  0.5031
 2   treatmentB        less     B     0 0.5052  0.5052
 3   treatmentC        less     C     0 0.0003  0.0003
   At least one                       0 0.6705  0.6705
            All                       0 0.0003  0.0003

-- Sample size per group:
        id ESS St.Dev q(0.1) q(0.5) q(0.9)
 1 control  65      0     65     65     65
 2       A  65      0     65     65     65
 3       B  65      0     65     65     65
 4       C  65      0     65     65     65
     Total 260      0    260    260    260

-- Scenarios:
     id treatmentA treatmentB treatmentC overall early
  1 001          0          0          1  0.3000     0
  2 221          2          2          1  0.2379     0
  3 021          0          2          1  0.1490     0
  4 201          2          0          1  0.1478     0
  5 220          2          2          0  0.0996     0
  6 020          0          2          0  0.0174     0
```

```
 7 200          2          0          0  0.0162     0
 8 101          1          0          1  0.0095     0
 9 011          0          1          1  0.0094     0
10 000          0          0          0  0.0090     0
11 111          1          1          1  0.0016     0
12 211          2          1          1  0.0011     0
13 121          1          2          1  0.0010     0
14 222          2          2          2  0.0003     0
15 210          2          1          0  0.0002     0
where 0 = no stop, 1 = efficacy stop, 2 = futility stop
```

Interpretation of Output

Compared to the adaptive design presented in Section 3.1, the fixed design shows a notable decrease in power for the effective arm (treatment C), from approximately 95% to 86%. The pairwise type I error rates decrease slightly (from approximately 1.9% to 1.2%).

As there is no early stopping, all simulated trials recruit to the maximum sample size of $N = 260$, and all arms receive equal sample sizes of 65 participants (SD = 0). In contrast, the adaptive design achieved a lower expected total sample size of 225.65 (SD = 44.04), with the most common scenario (54% of trials) being early futility stopping for both ineffective arms alongside an efficacy declaration for arm C. This illustrates the potential benefits of the adaptive design, which in this setting achieves higher power by dropping futile arms early and redistributing their sample size equally among the remaining arms, while maintaining comparable type I error control.

4 Summary of Results

The proportion of simulations which declared efficacy or futility for each intervention arm, any intervention arm, and all intervention arms (at any analysis/look) is displayed in Table B.1. Note that a "futility" decision at the maximum sample size could be interpreted as a low probability of finding a clinically important difference. These results were obtained using the `summary` command output. Through careful choice of the design parameters we were able to obtain a one-sided family-wise type I error rate of 5% under the global null, and $> 90\%$ power to detect a RR=0.4 under the scenarios explored. Ineffective arms (RR=1) had a futility decision in approximately 70% of the simulated trials, and arms with a large beneficial effect met the futility criteria in only a small proportion of simulated trials.

The run times for this example for a cluster and standard Windows desktop are provided in Table B.2. We encourage the user to initially simulate a small number

of trials (e.g., R = 25) to get a sense of the results and then increase the number of simulations once the tuning parameters and stopping rules have been refined to more precisely estimate type I error and power. A comparison with a fixed (non-adaptive) design using the same efficacy and futility parameter values illustrated the gains in power and expected sample size achieved by the adaptive design.

**Scenario**	**A**	**B**	**C**	**At least one**	**All**
**Efficacy decision**					
Null	0.016	0.019	0.020	0.050	0.001
One works	0.018	0.019	0.947	0.948	0.002
Better, Best	0.106	0.446	0.907	0.919	0.085
**Futility decision**					
Null	0.700	0.702	0.699	0.883	0.494
One works	0.708	0.714	0.011	0.842	0.011
Better, Best	0.398	0.121	0.013	0.428	0.007

Table B.1: Probability of declaring efficacy and futility (at any look) for each intervention arm, at least one arm, and all arms, for the overdispersed count outcome case study (Case Study 1).

5 Run times

The run times for Case study 1 are displayed in Table B.2

Scenario	Computer	Run time
Global Null	Cluster (1000 CPUs)	1.5 minute
One treatment works	Cluster (1000 CPUs)	1.5 minute
Better, Best	Cluster (1000 CPUs)	1.6 minute
Global Null	Standard Windows desktop (12 cores)	1.30 hours
One treatment works	Standard Windows desktop (12 cores)	1.42 hours
Better, Best	Standard Windows desktop (12 cores)	1.68 hours

Table B.2: Run times for Case study 1 for each scenario using 2 different computing set-ups

6 Bibliography

Curtin, F., H. Porchet, R. Glanzman, H. M. Schneble, V. Vidal, M.-L. Audoli-Inthavong, E. Lambert, and H. P. Hartung (2016). A placebo randomized controlled study to test the efficacy and safety of gnbac1, a monoclonal antibody for the treatment of multiple sclerosis – rationale and design. *Multiple Sclerosis and Related Disorders 9*, 95–100.

Gotmaker, R., M. J. Barrington, J. Reynolds, L. Trippa, and S. Heritier (2019). Bayesian adaptive design: the future for regional anesthesia trials? *Regional Anesthesia & Pain Medicine 44*(6), 617–622.

Hartung, H.-P., T. Derfuss, B. A. Cree, M. P. Sormani, K. Selmaj, J. Stutters, F. Prados, D. MacManus, H.-M. Schneble, E. Lambert, H. Porchet, R. Glanzman, D. Warne, F. Curtin, G. Kornmann, B. Buffet, D. Kremer, P. Küry, D. Leppert, T. Rückle, and F. Barkhof (2022). Efficacy and safety of temelimab in multiple sclerosis: Results of a randomized phase 2b and extension study. *Multiple Sclerosis Journal 28*(3), 429–440.
