# Supplementary material for: A Fast, Flexible Simulation Framework for Bayesian Adaptive Designs—The R Package BATSS
Source: Pharm Stat. 2026 Aug 10;25(5):e70109. doi: 10.1002/pst.70109 (PMC13456979; doi:10.1002/pst.70109)
Supplement: Supplementary file 3 [file PST-25-0-s002.docx]

# Appendix A - Detailed description of batss.glm function arguments, derived quantities/ingredients and workflow

# 1.0 Installation instructions

BATSS is available on the Comprehensive R Archive Network (CRAN) and Github, the latter of which provides a website with the package documentation and several tutorials
(https://batss-stable.github.io/BATSS/). BATSS can be installed using one of the following commands:

```
install.packages("BATSS") #CRAN
#install.packages("devtools")
devtools::install_github("batss-stable/BATSS") #Github
```

The BATSS package will install all required packages/dependencies with the exception of the R package INLA - required for calculation of posterior probabilities - as it is not available on CRAN. It should therefore be installed separately by the user prior to using BATSS (For instructions on how to install this package, we refer to <https://www.r-inla.org/download-install>. Note that instructions are operating-system specific with several binary builds available to Linux users, and that we advise installing its most up-to-date `testing' version instead of the (default) `stable' one).

Once installed the BATSS package is loaded with:

library(BATSS)

and simulations are performed using the batss.glm function.

# 2.0 batss.glm arguments

The batss.glm function has a number of arguments which the user can easily alter and several defaults are provided; these are described in Table A1 below. In Table A2 we provide a description of some of the common “ingredients” (derived quantities) that batss.glm calculates and can be used as inputs for the user-defined functions for adaptations (or evaluating efficacy or futility at a final look at the maximum sample size) and treatment arm allocations.

Table A1. Overview of the arguments/inputs into the batss.glm function

| **batss.glm function argument** | **Example(s)** | **Description** |
| --- | --- | --- |
| model | y ~ group  y ~ treatment  y ~ group + X1 + X2 | A model object (class: formula) indicating a symbolic description of the model to be fitted (using the same notation as in the lm and glm functions). This model is used to *simulate* *and fit* the data for the outcome variable and so the model object must be an appropriate formula for the inla function to handle. The names of the variables in the model may be chosen by the user. **However, the treatment allocation variable must be the first variable that is listed on the right-hand-side of the model equation.** |
| var | list(y = rnorm, group = alloc.balanced)  list(y=rbinom, treatment=alloc.simple)  list(y = rnbinom, group = alloc.balanced)  list(y = rnorm, group = alloc.simple, X=mvrnorm) | A list. Each entry corresponds to a variable described under 'model' (both the outcome and explanatory/predictor variables) and indicates, by means of a function name, how each variable is to be generated (using a function name for each variable). The list names **must** match the variable names as indicated under 'model' and the **first list element must correspond to the outcome.**  The variable corresponding to the treatment allocation has to be a factor with levels corresponding to the names indicated for prob0 (see below).  Existing random number generator functions from R libraries (e.g., rnorm, rbinom, rpois from R’s **stats** package) may be used, or user-defined functions (with function names chosen by the user); however, **INLA** must have an appropriate model available to analyse such outcome data. The function for allocating treatments (e.g. alloc.simple, alloc.balanced) will need to be a user-defined function and will probably require a line of code to normalise the probabilities (to sum to one), unless it is already specified in the user-defined RAR function (see below). |
| var.control | list(y=list(sd=5))  #use for y=rnorm  list(y=list(size=1))  #use for y=rbinom  list(y = list(size = 1/2))  #use for y = rnbinom  list(y=list(sd=5),X=list(mu=c(0,5), Sigma=matrix(c(1,0.5,0.5,1),2,2)))  #use for y = rnorm, X=mvrnorm | An optional list of control/input parameters for functions in 'var', such as nuisance parameters. The names of the list items need to correspond with the names of 'var'. Each element is another list with the names of these elements corresponding to the parameter names that are input into the functions specified in 'var'.  This list does not need to contain all variables listed in the model (only those that need additional parameters specified). The default = NULL (e.g. use this option for a Poisson likelihood family if no additional parameters are needed for the predictors’ functions, and y=rpois in the 'var' list).  If the treatment allocation function requires inputs beyond “m” and “prob” (defined below), then these must be specified here too (otherwise there is no need to specify anything for the treatment allocation function). |
| family | “gaussian” “binomial” “nbinomial”  “poisson” | A character string indicating the name of the conditional distribution (likelihood family), as described in the package **INLA**, that is to be used to **fit** the described ‘model’ to the data. Default set to “gaussian”.  Options that are currently available are: “gaussian”,  “binomial”, “nbinomial”, “poisson” |
| link | “identity” “logit” “log”  “probit”  “robit”  “cauchit”  “loglog”  “cloglog” | A character string describing the link function to be used in the model to relate the outcome to the set of predictors: "identity", "logit", "log", “probit”, “robit”, “cauchit”, “loglog” and “cloglog” are the currently available options. Default is set to “identity”. This is used in both simulating data and fitting the described ‘model’ to the data. |
| beta | c(20,3,3,5)  c(log(0.4/(1-0.4)), log(1), log(1), log(1)) | A numerical vector of parameter values for the regression parameters to simulate from as the “true values” for the linear predictor. Its length has to match the number of columns of the X matrix induced by the formula indicated under 'model' (check ?model.matrix). i.e., it must include a parameter for each intervention arm (including an intercept) and any covariates (and corresponding dummy variables for factors). The first value of beta is the intercept / mean response in control (on the linear predictor scale, e.g., log odds) (for covariates = 0), and the subsequent values of beta that correspond to the intervention arms are the change from control to the intervention arm (treatment effect) on the linear predictor scale; the remaining beta values are for the covariate effects (on the linear predictor scale). |
| which | c(2:4)  c(2,3) | A numerical vector indicating the position (indices) of the “target parameters” in ‘beta’ (i.e., the treatment/intervention arms of interest; doesn’t include the control or other covariates, just the regression parameters of interest for treatment effects). |
| alternative | c("greater")  c(“less”) | A vector of strings providing the one-sided direction of the alternative hypothesis corresponding to each target parameter (treatment effect) indicated under 'which'. Options are “greater” (default) or “less”. If the vector is of length 1, the same direction will be used for all target parameter tests.  These are used in conjunction with ‘deltas’ (see below) where the hypothesis is evaluated in the same direction for both efficacy and futility decisions (and RAR) for a target parameter  (i.e. Pr($\beta_{g}$ > delta.eff), Pr($\beta_{g}$> delta.fut) if alternative = “greater” OR  Pr($\beta_{g}$ < delta.eff), Pr($\beta_{g}$ < delta.fut) if alternative = “less” for intervention arm *g*). |
| R | 1000  10000  1:25 | A scalar corresponding to the number of Monte Carlo trials or a vector of natural numbers to be used as seeds (check ?set.seed) for the different Monte Carlo trials (the vector length will thus correspond to the number of Monte Carlo trials). If a scalar is used, then seeds 1 to R are used. Default is 10,000. |
| N | 260 | A scalar indicating the maximum sample size for the trial |
| interim | i) list(recruited=list(m0=100,m=40))  OR ii) list(recruited=c(100,140, 180, 220))   OR   iii) NA | A list of parameters related to the interim analysis. Currently, only the parameter ‘recruited’ is available, and consists of a vector of integers in increasing order that provides the total sample size of **complete** observations (completed primary outcome follow up) at each adaptive analysis point, with the last look (at maximum sample size) excluded.  The user may either provide i) a list with components m0, and m (defined below; these names must be used), or ii) a vector of integers (in increasing size).  m0 provides the timing of the first adaptive analysis (sample size at first adaptive analysis, in terms of complete observations).  m provides the spacing between the adaptive analyses (increment in numbers of patients with complete observations between looks).  If the interim argument is specified using m0 and m, the (adaptive) analysis times are generated using the *seq* function in R; the last value that is generated in the vector by *seq* is replaced by N (to potentially perform a final analysis at the maximum sample size if the trial does not stop early).  If the user provides a vector of increasing size (option ii) above), then N will be added on to the end of this vector (last potential look at the data)  If interim is set to NA, no interim analysis will take place and a fixed design will be used. |
| prob0 | c(A=1/4,B=1/4,C=1/4,D=1/4)  c(A=1,B=1,C=1,D=1)  c(control = 2, A=1, B=1) | A named vector with the initial allocation probabilities/ratios for the burn-in period for RAR. If RAR=NULL then these probabilities/ratios will be used throughout (fixed allocation probabilities). Names need to correspond to the levels of the treatment allocation variable. **The reference/control arm must be listed first (i.e. must be the first component of the named vector).**  Note: The user-defined treatment allocation function (e.g., alloc.simple) should have a line of code to normalise the allocation probabilities (to sum to one). If RAR=NULL, and if arm dropping is permitted, then prob0 will be reweighted to sum to 1 via the treatment allocation function. If RAR is used (i.e., RAR != NULL) then normalisation of the probabilities (to sum to one) can occur within the user-defined RAR function code. |
| delta.eff | 0  3  c(NA,0,0,0)  c(rep(NA,13), 0) | A scalar or vector defining the clinically meaningful treatment effect value(s) of interest (on the linear predictor scale; e.g., log odds with link='logit' and log fold change with link='log') that beta must exceed to declare efficacy for an arm at each analysis.   delta.eff may be a vector with length matching the number of looks/analyses (including a final look at the maximum sample size) if you want delta.eff to change across the looks, or it can be a scalar where the same value is used at each analysis. Use NAs in this vector if you don’t want to stop for efficacy at a particular interim analysis (at the corresponding analysis point(s)).  The same delta.eff value is used to compare each intervention to the control, at a particular look (i.e., separate delta.eff values can’t be used for different comparisons at the same analysis point).  As an example, for a binary outcome where the control arm rate/proportion for an outcome is p0, and you’re interested in seeing at least a 10% absolute increase in the proportion in an intervention arm, i.e., p0 + 0.1, then for a model using a logit link, the delta.eff would be: delta.eff=log(((p0+0.1)/(1-(p0+0.1)))/(p0/(1-p0))).  Here we would be interested in the quantity  Pr(β > delta.eff \| data) (i.e. posterior probability β > delta.eff)  Alternatively, if one wanted to stop for efficacy if there was a high posterior probability that the OR<0.8, then delta.eff = log(0.8) here.  The default value for delta.eff is 0. |
| eff.arm | eff.arm.simple  eff.arm.infofract  efficacy.arm.fun | A function name that refers to a user-defined function indicating how to evaluate if efficacy has been achieved at a given analysis point/look given the information (ingredients) available at that stage for a given target parameter (i.e., efficacy is evaluated for each (active) intervention arm using this function). This allows individual arms to be stopped for efficacy (early selection). More than one arm can be stopped at a particular adaptive analysis. If efficacy is achieved for that arm then randomisation to that arm is immediately terminated (arm is dropped). Alternatively, the user may choose to stop the trial as soon as an arm has demonstrated efficacy over the control (through the specification of the ‘eff.trial’ function; see below).    This function is also used to evaluate efficacy for an arm at the maximum sample size (if it is not stopped early), and so the user must provide a function for this argument if they wish to evaluate efficacy in the trial. If eff.arm = NULL then efficacy evaluations will never occur at any look in the trial.    The output of this expression must be a logical (T/F) of length 1.  If you don’t want to permit early stopping for efficacy, set delta.eff = NA for all of the interim analysis looks (and provide a value for delta.eff for a final analysis/look). Similarly, use NAs in this vector if you don’t want to stop for efficacy at a particular interim analysis (at the corresponding analysis point(s)). |
| eff.arm.control | list("b.eff"=0.012, "p.eff"=1.4)  list(b.eff = 0.045)  list(b = 0.975) | An optional list of parameters that are to be used as inputs in the function defined in 'eff.arm', such as the posterior probability threshold for superiority/efficacy. |
| eff.trial | eff.trial.all  eff.trial.any | A function name that refers to a user-defined function (with the ingredient/input “eff.target”; see below for definition) which indicates if the trial can be stopped for efficacy given the output from the function specified in 'eff.arm' on all target parameters (across all adaptive analyses/looks to-date). The output of this expression must be a logical of length one (T/F). When eff.trial=NULL (default), the trial stops for efficacy only when **all** target parameters (intervention arms) are found to be effective (have been stopped for efficacy). |
| eff.trial.control |  | An optional list of parameters that are to be used in the function defined in 'eff.trial' |
| delta.fut | 3  log(0.8)  c(rep(log(0.8),5), 0)  c(NA, rep(0,5)) | A scalar or vector defining the clinically meaningful treatment effect value(s) of interest (on the linear predictor scale, e.g., log odds with link='logit' and log fold change with link='log') that, if beta can’t exceed (with a certain posterior probability), then the intervention arm will stop for futility/lack-of-benefit (either at an interim analysis or at the maximum sample size if it hasn’t stopped earlier).  Note that only one lack-of-benefit statistical trigger/adaptation can currently be implemented (e.g., futility OR inferiority; both aren’t currently able to be simultaneously evaluated).  delta.fut may be a vector with length matching the number of looks/analyses (including a final look at the maximum sample size) if you want delta.fut to change across the looks, or it can be a scalar where the same value is used at each analysis. Use NAs in this vector if you don’t want to stop for futility at a particular interim analysis (at the corresponding analysis point(s)).  The same delta.fut value is used to compare each intervention to the control, at a particular look (i.e., separate delta.fut values can’t be used for different comparisons at the same analysis point).  As an example, for a binary outcome, if one wanted to stop for futility if there was a low posterior probability the OR<0.8, then delta.fut = log(0.8).  Here we would be interested in the quantity  Pr(β < delta.fut \| data) (i.e. posterior probability β < delta.fut).  Default values: if no delta.fut value is specified (and a delta.eff value is specified) then delta.fut = delta.eff. If neither delta.fut or delta.eff are specified, then the default is delta.fut = 0. |
| fut.arm | futility.arm.simple | A function name referring to a user-defined function indicating how to evaluate if futility/lack-of-benefit has been reached at a given analysis point/look given the information (ingredients) available at that stage for a given target parameter (i.e., futility is evaluated for each (active) intervention arm using this function). This allows individuals arms to be stopped for lack-of-benefit. This function is also used to evaluate lack-of-benefit for an arm at the maximum sample size (final look) if it is not stopped early, and so the user must provide a function for this argument. If fut.arm = NULL then futility evaluations will never occur at any look in the trial.  The output of this expression must be logical (T/F) of length 1.  More than one arm can be stopped at an adaptive analysis. If an arm is dropped for futility/lack-of-benefit then randomisation to that arm is immediately terminated.  If you don’t want to permit early stopping for futility, set delta.fut=NA for the interim analysis looks. |
| fut.arm.control | list("b.fut"=0.05)  list(b.fut = 0.8, p.fut = 1.8) | An optional list of parameters that are used as inputs in the function defined in 'fut.arm', such as the posterior probability threshold for futility. |
| fut.trial | fut.trial.all | A function name that refers to a user-defined function (with ingredient “fut.target”; see below for definition) which indicates if the trial can be stopped for futility given the output of the function specified in fut.arm on all target parameters (across all adaptive analyses/looks to-date). The output of this expression must be logical of length one (T/F). When fut.trial=NULL (default), the trial stops for futility when **all** of the target parameters (intervention arms) have been dropped for futility/lack-of-benefit. |
| fut.trial.control |  | An optional list of parameters that are to be used as inputs in the function defined in 'fut.trial' |
| RAR | RAR.trippa  RAR.optimal  rar.fun | A function name that refers to a user-defined function that indicates how the response-adaptive randomisation probabilities corresponding to each treatment group, control/reference group included, are defined. If RAR=NULL (default), fixed probabilities (defined in prob0) of being randomised to each (active/remaining) treatment arm are used.  This function could be alternatively used for calculating randomisation probabilities/allocation ratios which may change depending on the number of active (intervention) arms remaining in the trial (e.g., see the BATSS function RAR.optimal). |
| RAR.control | list("gamma"=3, "eta"=1.4, "nu"=1/10) | An optional list of control parameters that are used in the function defined in 'RAR'. |
| delta.RAR | 0 | A scalar or vector defining the clinically meaningful treatment effect value(s) of interest (on the linear predictor scale, e.g., log odds with link='logit' and log fold change with link='log') that beta is compared against when calculating the posterior probabilities to be used for RAR, i.e. pr(beta > delta.RAR \| data) or pr(beta < delta.RAR \| data). A default value of 0 is used.  delta.RAR may be a vector with length matching the number of looks (where the delta.RAR value for the last potential look at the maximum sample size is ignored as no updating of randomisation probabilities are performed then) if you want delta.RAR to change across the looks, or it can be a scalar where the same value is used at each analysis. The same delta.RAR value is used to compare each intervention to the control, at a particular look (i.e., separate delta.RAR values can’t be used for different comparisons at the same analysis point). |
| H0 | FALSE  TRUE | A logical indicating whether the simulation should also consider the case with all target (beta) parameters set to 0 (global null). Default is H0= TRUE |
| computation | “parallel” “sequential” | A character string indicating how the computation should be performed. Possibilities are “parallel” or “sequential”, with default computation = “parallel”, meaning that computation is split between mc.cores. |
| mc.cores | 12  getOption("mc.cores", 3L)  detectCores()-1 | An integer indicating how many cores to use when computation='parallel' (Default to 3 if no global option was set). A good option might be to use detectCores()-1 |
| extended | 0  1  2 | An integer indicating the type of results to be returned (simulation output to be stored). 0 (default) provides just the summary statistics/operating characteristics. 1 adds the results of each Monte Carlo trial (overall trial results and breakdown by interim analysis). 2 additionally returns the datasets for each of the Monte Carlo trials.  BATSS::batss.combine requires that extended >0 as the function needs to merge results of different sets of seeds. |
| … |  | Additional arguments to control fitting in INLA |

Table A2. Description of the common “ingredients” (derived quantities) that batss.glm calculates at each look and can be used as inputs to the user-defined functions for the adaptations, group allocations, and assessing efficacy/futility at a final look (at the maximum sample size)

| **Ingredient/quantity (inputs for user-defined functions for adaptations)** | **Description** |
| --- | --- |
| posterior | Depending on the context - efficacy, futility or RAR, posterior may correspond to different probabilities and consist of different lengths.  Efficacy: Posterior probability of efficacy of an “active” intervention (intervention that’s still in the study), incorporating the delta.eff. It consists of a numerical scalar for the target parameter (active intervention) that is currently being evaluated at a particular look.  Futility: Posterior probability of futility of an “active” intervention, incorporating the delta.fut. It consists of a numerical scalar for the target parameter (active intervention) that is currently being evaluated at a particular look. This will be the same value as for efficacy if delta.fut = delta.eff.  RAR: Posterior probability of efficacy for each “active” intervention, incorporating the delta.RAR. This is a vector of length = number of remaining active intervention arms at a given look. |
| n | The number of participants that have completed follow-up in each treatment arm at the look of interest; includes control and intervention arms and both active and dropped (inactive) arms. i.e., sum(n) is the total number of participants that have completed follow-up to-date in the trial. This is a vector of length = number of treatment arms (initially included) in the trial. |
| active | A logical vector (i.e. consists of T/F) that indicates which treatment arms are still active/remaining in the trial at a given look and includes both control and intervention arms. This is a vector of length = number of treatment arms (initially included) in the trial. |
| ref | A logical vector (i.e. consists of T/F) that indicates which treatment is the reference treatment (i.e. control arm). This is a vector of length = number of treatment arms in the (beginning of the) trial. The reference treatment is typically the first element of this vector (i.e., first element = T, remaining elements = F). |
| prob | A numerical vector of length = number of active arms (including control) that is output from the user-defined RAR function to provide the randomisation ratios (to be used in the treatment/group allocation functions). This vector is typically unnormalised (normalisation to sum to 1 occurs in the treatment allocation function (e.g., alloc.simple); make sure a line is included in the treatment allocation function described in the “var” argument in batss.glm (e.g., alloc.simple) to do this). This vector has names for each column corresponding to the treatment arm names provided in prob0. If RAR = NULL then prob = prob0 (with appropriate subsetting for arms that have been dropped). |
| m | As defined previously (i.e. total number of participants that will have been recruited and completed follow-up since the last interim analysis/for the next look). |
| N | As defined previously (i.e. maximum total sample size for the trial); specified as an argument in batss.glm |
| eff.target | A logical vector (i.e. consists of T/F) that indicates whether an intervention arm has been dropped for efficacy (across all looks, current or previous); length = number of interventions / target parameters |
| fut.target | A logical vector (i.e. consists of T/F) that indicates whether an intervention arm has been dropped for futility/lack-of-benefit (across all looks, current or previous); length = number of interventions / target parameters |

# 3.0 **batss.glm workflow**

For each simulated trial, at the beginning of each analysis period (trial “stage”), *m* simulated participants are randomly allocated to arms that are currently active (*m0* patients for the first stage). This is achieved by using allocation probabilities that can be calculated using RAR or fixed allocation probabilities. The user-defined functions are used to generate outcomes for each participant, and the user-specified model is fit using R-INLA to generate marginal posteriors for the target parameters (i.e., treatment effects). Posterior probabilities for efficacy and futility are then calculated for each pairwise comparison of control to the currently active interventions (i.e., those that have not been dropped), using the “deltas” (clinically important effects). Interventions may then be dropped for efficacy or futility if they have met the user-defined stopping rules (more than one intervention may be dropped at an interim analysis) and the entire trial may be stopped if the trial stopping rules have been met. If the trial does not stop, and RAR is selected for the design (or an arm has been dropped), then the randomisation probabilities are updated and the trial moves to the next analysis period. This process is repeated until the trial has met the user-defined stopping rules or the maximum sample size has been reached.
